# Crystal structure of methyl 3′-benzamido-4′-(4-meth­oxy­phen­yl)-1′-methyl­spiro­[indeno­[1,2-*b*]quinoxaline-11,2′-pyrrolidine]-3′-carboxyl­ate

**DOI:** 10.1107/S2056989016012469

**Published:** 2016-08-05

**Authors:** Kuppan Chandralekha, Adukamparai Rajukrishnan Sureshbabu, Deivasigamani Gavaskar, Srinivasakannan Lakshmi

**Affiliations:** a Research Department of Physics, S. D. N. B. Vaishnav College for Women, Chromepet, Chennai 600 004, India; b Department of Organic Chemistry, University of Madras, Guindy Campus, Chennai 600 025, India

**Keywords:** crystal structure, indeno­quinoxaline, pyrrolidine, spiro pyrrolizidine, N—H⋯N inter­action

## Abstract

In the title compound, C_35_H_30_N_4_O_3_, the spiro C atom connects the five-membered pyrrolidine ring and the indeno­quinoxaline ring system. The pyrrolidine ring adopts a twist conformation. An intra­molecular N—H⋯N inter­action between the amino group and the pyrazine ring is observed. In the crystal, mol­ecules are linked by a pairs of C—H⋯O hydrogen bonds, forming inversion dimers.

## Abstract 2

In the title compound, the mean plane through pyrrolidine ring is approximately orthogonal to the mean plane of the cyclo­pentane ring, making a dihedral angle of 88.78 (10)°. An intra­molecular N—H⋯N inter­action is observed. The crystal packing features C—H⋯O hydrogen bonds.

## Chemical context   

Spiro pyrrolidine derivatives act as potential anti­leukemic (Abou-Gharbia & Doukas, 1979 ▸), anti­convulsant (Jiang *et al.*, 2006 ▸), anti­viral (Lundahl *et al.*, 1972 ▸) and anti-inflammatory (Hussein & Abdel-Monem, 2011 ▸) agents. Indeno­quinoxaline derivatives possess anti­metabolism properties (Sehlstedt *et al.*, 1998 ▸) and find applications in dyes. They are also used as building blocks for the synthesis of organic semiconductors (Gazit *et al.*, 1996 ▸).
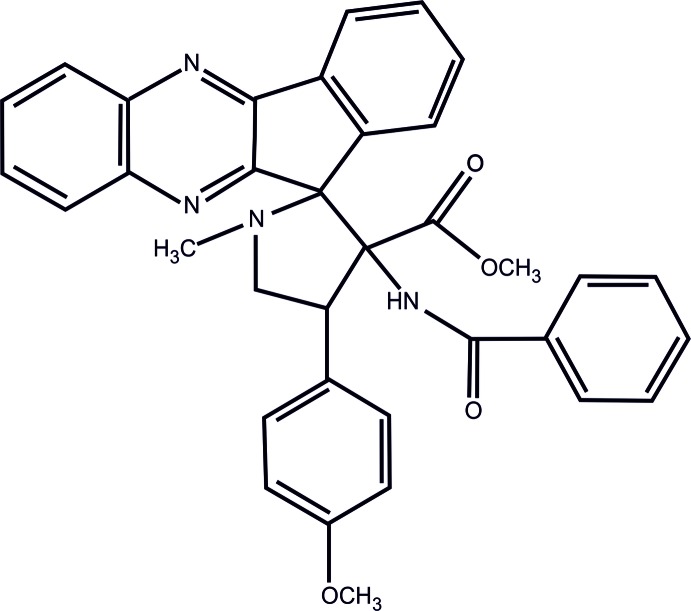


The synthesis of di­spiro­indeno­quinoxaline pyrrolidine derivatives has been achieved by one-pot four-component 1,3-dipolar cyclo­addition reaction (Suresh Babu & Raghunathan, 2008 ▸) while ninhydrin-based one-pot four-component condensation reaction yielded novel alkyl­spiro­[indeno­[1,2-*b*]quinoxaline-11,3′-pyrrolizine]-2′-carboxyl­ate derivatives (Karsalary *et al.*, 2010 ▸). A series of original spiro­pyrrolizidine derivatives was synthesized by a one-pot three-component [3 + 2] cyclo­addition reaction; these exhibit extensive hydrogen bonding in the crystalline state (Haddad *et al.*, 2015 ▸).

## Structural commentary   

**Figure 1:**
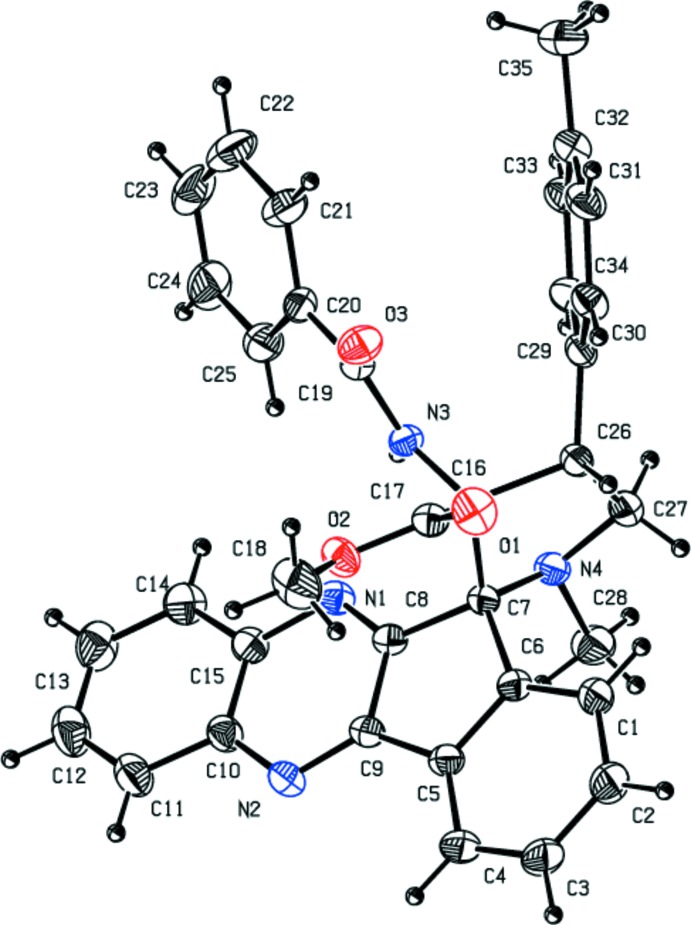
The mol­ecular structure of the title compound, with displacement ellipsoids drawn at the 30% probability level. H atoms are shown as small arbitrary radius.

**Figure 2:**
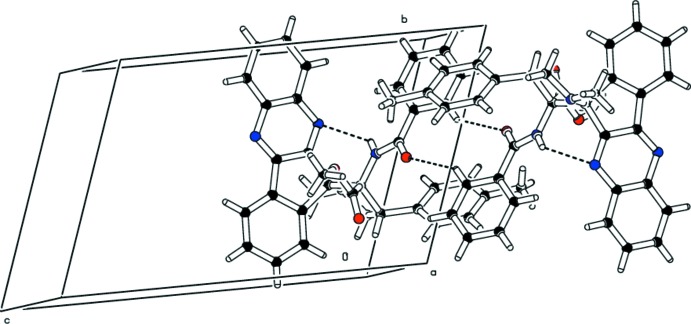
Partial packing diagram for the title compound, showing the formation of dimers *via* C—H⋯O inter­actions (dashed lines). The intra­molecular N—H⋯N hydrogen bond is also shown (dashed lines).

In the title compound (Fig. 1 ▸), the four-fused-ring system of the 11*H*-indeno­[1,2-*b*]quinoxaline unit is approximately planar and forms a dihedral angle of 59.16 (7)° with the C29–C34 methyl­benzene ring. The methyl-substituted C7/C16/C26/C27/N4 pyrrolidine ring is in a twist conformation with puckering parameters *Q*(2) = 0.4238 (18) Å and φ = 215.8 (2)°. The mean plane through the C7/C16/C26/C27/N4 pyrrolidine ring is approximately orthogonal to the mean plane of the C5–C9 cyclo­pentane ring, subtending a dihedral angle of 88.78 (10)°. The mean plane of the pyrrolidine ring makes a dihedral angle of 70.33 (10)° with the attached benzene ring. The sum of bond angles around nitro­gen atom of the pyrrol­idine ring (337.11°) is in agreement with *sp* ^3^ hybridization. An intra­molecular N—H⋯N hydrogen bond stabilizes the mol­ecular conformation (see Table 1 ▸ and Fig. 2 ▸).

## Supra­molecular features   

In the crystal, symmetry-related enanti­omeric mol­ecules are linked through pairs of C—H⋯O inter­actions (Table 1 ▸), forming dimers with an 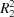
(10) graph-set motif. This inter­molecular C—H⋯O hydrogen bond, along with the intra­molecular N—H⋯N inter­action, plays an important role in stabilizing the packing of the mol­ecules.

## Database Survey   

A search of the Cambridge Structural Database (Version 5.36, last update May 2015; Groom *et al.*, 2016 ▸) revealed that the number of compounds containing a pyrrolidine ring is 2420 and a quinoxaline unit is 1265. Out of these entries, only 14 compounds were found to possess both pyrrolidine and quinoxaline ring systems. The geometry of the pyrrolidine ring of the title compound compares well with those reported for similar structures, for example, 4-ferrocenyl-1-methyl-3-benzoyl­spiro­[pyrrolidine-2,11′-indeno­[1,2-*b*]-quinoxaline (refcode: EDUSED; Vijayakumar *et al.*, 2012 ▸). The bond lengths and bond angles of quinoxalin unit are in good agreement with reported values of a related structure (refcode: MOKNUX; Chandralekha *et al.*, 2014 ▸). The N—H⋯N hydrogen bond is a rare occurrence in these type of compounds (refcodes: IFOQIF, NINVEN, NIPDUN, LOSKAH, HOWCIH, BENDEF, CEFDOI, EDUSED).

## Synthesis and crystallization   

A mixture of ninhydrin (1 mmol) and 1,2-phenyl­enedi­amine (1 mmol) were stirred for 15 min in methanol (10 mL). Then, to this was added a solution of 4-(4-methyl­benzyl­idene)-2-phenyl-4*H*-oxazole-5-one (1 mmol) and sarcosine (1 mmol) in methanol (10 mL). The reaction mixture was refluxed for 16–18 h and the progress of the reaction was monitored by TLC. After the completion of the reaction as evidenced by TLC, the excess solvent was removed under vacuum and the crude product was purified by column chromatography using a mixture of petroleum ether and ethyl acetate as eluent (4:1). Single crystals suitable for the X-ray diffraction analysis were obtained by slow evaporation of the solvent at room temperature.

## Refinement   

Crystal data, data collection and structure refinement details are summarized in Table 2 ▸. All H atoms were placed in calculated positions, with C—H = 0.93–0.98 and N—H = 0.86 Å, and were refined using a riding-model approximation, with *U*_iso_(H) = 1.2*U*_eq_(C, N) or 1.5*U*_eq_(C) for methyl H atoms. A rotating model was applied to the methyl groups.

## Supplementary Material

Crystal structure: contains datablock(s) I. DOI: 10.1107/S2056989016012469/is5458sup1.cif

Structure factors: contains datablock(s) I. DOI: 10.1107/S2056989016012469/is5458Isup2.hkl

Supporting information file. DOI: 10.1107/S2056989016012469/is5458Isup3.cml

CCDC reference: 1497294

Additional supporting information: crystallographic information; 3D view; checkCIF report

## Figures and Tables

**Table 1:** Hydrogen-bond geometry (Å, °)

*D*—H⋯*A*	*D*—H	H⋯*A*	*D*⋯*A*	*D*—H⋯*A*
N3—H3⋯N1	0.86	2.27	2.8107 (18)	121
C21—H33⋯O3^i^	0.93	2.54	3.347 (2)	146

Symmetry code: (i) 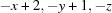.

**Table 2:** Experimental details

Crystal data	
Chemical formula	C_35_H_30_N_4_O_3_
*M*_r_	554.63
Crystal system, space group	Triclinic, *P* 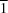
Temperature (K)	293
*a*, *b*, *c* (Å)	10.1194 (4), 10.8066 (4), 14.9948 (6)
α, β, γ (°)	110.57 (2), 97.10 (2), 106.17 (2)
*V* (Å^3^)	1429.1 (4)
*Z*	2
Radiation type	Mo *K*α
μ (mm^−1^)	0.08
Crystal size (mm)	0.35 × 0.30 × 0.25

Data collection	
Diffractometer	Bruker Kappa APEXII CCD
Absorption correction	Multi-scan (*SADABS*; Bruker, 2004 ▸)
*T*_min_, *T*_max_	0.719, 0.746
No. of measured, independent and observed [*I* > 2σ(*I*)] reflections	42242, 8042, 4733
*R*_int_	0.034
(sin θ/λ)_max_ (Å^−1^)	0.717

Refinement	
*R*[*F* ^2^ > 2σ(*F* ^2^)], *wR*(*F* ^2^), *S*	0.046, 0.140, 1.03
No. of reflections	8042
No. of parameters	383
H-atom treatment	H-atom parameters constrained
Δρ_max_, Δρ_min_ (e Å^−3^)	0.23, −0.20

Computer programs: *APEX2* and *SAINT* (Bruker, 2004 ▸), *SHELXS97* (Sheldrick, 2008 ▸), *SHELXL2014* (Sheldrick, 2015 ▸), *ORTEP-3 for Windows* (Farrugia, 2012 ▸), *PLATON* (Spek, 2009 ▸) and *publCIF* (Westrip, 2010 ▸).

## supplementary crystallographic information

### Crystal data

**Table:**

C_35_H_30_N_4_O_3_	*Z* = 2
*M**_r_* = 554.63	*F*(000) = 584
Triclinic, *P*1	*D*_x_ = 1.289 Mg m^−^^3^
*a* = 10.1194 (4) Å	Mo *K*α radiation, λ = 0.71073 Å
*b* = 10.8066 (4) Å	Cell parameters from 42296 reflections
*c* = 14.9948 (6) Å	θ = 2.1–30.6°
α = 110.57 (2)°	µ = 0.08 mm^−^^1^
β = 97.10 (2)°	*T* = 293 K
γ = 106.17 (2)°	Block, colourless
*V* = 1429.1 (4) Å^3^	0.35 × 0.30 × 0.25 mm

### Data collection

**Table:**

Bruker Kappa APEXII CCD diffractometer	4733 reflections with *I* > 2σ(*I*)
Radiation source: graphite	*R*_int_ = 0.034
Bruker axs kappa axes2 CCD Diffractometer scans	θ_max_ = 30.6°, θ_min_ = 2.1°
Absorption correction: multi-scan (SADABS; Bruker, 2004)	*h* = −14→14
*T*_min_ = 0.719, *T*_max_ = 0.746	*k* = −15→15
42242 measured reflections	*l* = −20→20
8042 independent reflections	

### Refinement

**Table:**

Refinement on *F*^2^	Hydrogen site location: inferred from neighbouring sites
Least-squares matrix: full	H-atom parameters constrained
*R*[*F*^2^ > 2σ(*F*^2^)] = 0.046	*w* = 1/[σ^2^(*F*_o_^2^) + (0.0522*P*)^2^ + 0.3893*P*] where *P* = (*F*_o_^2^ + 2*F*_c_^2^)/3
*wR*(*F*^2^) = 0.140	(Δ/σ)_max_ < 0.001
*S* = 1.03	Δρ_max_ = 0.23 e Å^−^^3^
8042 reflections	Δρ_min_ = −0.20 e Å^−^^3^
383 parameters	Extinction correction: SHELXL2014 (Sheldrick, 2015), Fc^*^=kFc[1+0.001xFc^2^λ^3^/sin(2θ)]^-1/4^
0 restraints	Extinction coefficient: 0.0083 (13)

### Special details

**Table:**

Geometry. All esds (except the esd in the dihedral angle between two l.s. planes) are estimated using the full covariance matrix. The cell esds are taken into account individually in the estimation of esds in distances, angles and torsion angles; correlations between esds in cell parameters are only used when they are defined by crystal symmetry. An approximate (isotropic) treatment of cell esds is used for estimating esds involving l.s. planes.

### Fractional atomic coordinates and isotropic or equivalent isotropic displacement parameters (Å^2^)

**Table:**

	*x*	*y*	*z*	*U*_iso_*/*U*_eq_	
O3	0.90953 (13)	0.46770 (13)	0.11738 (9)	0.0534 (3)	
C18	1.0071 (2)	0.4247 (3)	0.35278 (16)	0.0756 (7)	
H1A	0.9742	0.3463	0.3705	0.113*	
H1B	1.0612	0.5078	0.4107	0.113*	
H1C	1.0657	0.4053	0.3077	0.113*	
N1	0.65819 (15)	0.65584 (13)	0.34701 (9)	0.0400 (3)	
N2	0.73382 (15)	0.62468 (15)	0.52637 (9)	0.0444 (3)	
N3	0.72086 (13)	0.50088 (13)	0.17371 (9)	0.0352 (3)	
H3	0.6741	0.5573	0.1898	0.042*	
N4	0.44863 (14)	0.37083 (14)	0.20107 (9)	0.0401 (3)	
O2	0.88730 (12)	0.44674 (13)	0.30692 (8)	0.0480 (3)	
C8	0.63890 (16)	0.53406 (16)	0.35130 (10)	0.0350 (3)	
C16	0.67525 (16)	0.37164 (15)	0.18823 (10)	0.0334 (3)	
C29	0.60006 (17)	0.25131 (16)	−0.00058 (10)	0.0375 (3)	
O1	0.81760 (14)	0.22640 (13)	0.19250 (10)	0.0585 (3)	
C6	0.57491 (17)	0.29247 (16)	0.31890 (11)	0.0377 (3)	
C7	0.58109 (16)	0.39113 (15)	0.26483 (10)	0.0344 (3)	
C9	0.67140 (16)	0.51875 (17)	0.44116 (11)	0.0367 (3)	
C5	0.62551 (17)	0.36968 (17)	0.42023 (11)	0.0384 (3)	
C26	0.56904 (17)	0.24648 (16)	0.09437 (10)	0.0379 (3)	
H15	0.5757	0.1587	0.0964	0.045*	
C10	0.76469 (18)	0.75469 (17)	0.52334 (12)	0.0435 (4)	
C17	0.80205 (17)	0.33770 (17)	0.22560 (11)	0.0396 (4)	
C15	0.72413 (18)	0.77054 (17)	0.43540 (12)	0.0418 (4)	
C19	0.83285 (17)	0.53765 (17)	0.13631 (11)	0.0395 (4)	
C20	0.85645 (17)	0.66951 (17)	0.11898 (12)	0.0432 (4)	
C30	0.67957 (18)	0.17659 (17)	−0.04772 (11)	0.0437 (4)	
H21	0.7072	0.1167	−0.0238	0.052*	
C14	0.7555 (2)	0.90641 (19)	0.43761 (14)	0.0551 (5)	
H22	0.7261	0.9184	0.3809	0.066*	
C1	0.5266 (2)	0.14754 (18)	0.28220 (13)	0.0506 (4)	
H23	0.4945	0.0941	0.2148	0.061*	
C32	0.6805 (2)	0.27734 (19)	−0.16772 (12)	0.0497 (4)	
C27	0.42273 (18)	0.25135 (19)	0.10854 (12)	0.0477 (4)	
H25A	0.3725	0.2637	0.0547	0.057*	
H25B	0.3662	0.1646	0.1112	0.057*	
C34	0.55824 (19)	0.33612 (18)	−0.04027 (12)	0.0469 (4)	
H26	0.5017	0.3852	−0.0113	0.056*	
C31	0.7190 (2)	0.18931 (19)	−0.13021 (12)	0.0504 (4)	
H27	0.7724	0.1375	−0.1608	0.060*	
C33	0.5985 (2)	0.3496 (2)	−0.12196 (12)	0.0523 (4)	
H28	0.5698	0.4085	−0.1465	0.063*	
C11	0.8396 (2)	0.8756 (2)	0.60964 (14)	0.0585 (5)	
H29	0.8674	0.8665	0.6678	0.070*	
C4	0.6250 (2)	0.30391 (19)	0.48471 (13)	0.0493 (4)	
H30	0.6584	0.3566	0.5522	0.059*	
C25	0.8142 (2)	0.77785 (19)	0.17246 (14)	0.0570 (5)	
H31	0.7671	0.7694	0.2207	0.068*	
C3	0.5742 (2)	0.1597 (2)	0.44692 (14)	0.0569 (5)	
H32	0.5720	0.1137	0.4891	0.068*	
C21	0.9233 (2)	0.6814 (2)	0.04555 (15)	0.0594 (5)	
H33	0.9513	0.6086	0.0084	0.071*	
C28	0.3265 (2)	0.3608 (2)	0.24324 (14)	0.0590 (5)	
H34A	0.2988	0.2748	0.2528	0.088*	
H34B	0.2492	0.3615	0.1995	0.088*	
H34C	0.3506	0.4395	0.3053	0.088*	
C23	0.9084 (2)	0.9101 (3)	0.08298 (19)	0.0767 (7)	
H35	0.9273	0.9917	0.0714	0.092*	
C12	0.8717 (2)	1.0055 (2)	0.60860 (16)	0.0670 (6)	
H36	0.9227	1.0847	0.6659	0.080*	
C13	0.8290 (2)	1.0212 (2)	0.52266 (16)	0.0657 (6)	
H37	0.8507	1.1110	0.5232	0.079*	
C2	0.5264 (2)	0.0822 (2)	0.34684 (14)	0.0596 (5)	
H38	0.4934	−0.0156	0.3223	0.071*	
C35	0.7294 (3)	0.2971 (3)	−0.25442 (15)	0.0755 (7)	
H39A	0.6599	0.3178	−0.2908	0.113*	
H39B	0.7413	0.2124	−0.2962	0.113*	
H39C	0.8183	0.3738	−0.2315	0.113*	
C24	0.8410 (3)	0.8988 (2)	0.15508 (17)	0.0717 (6)	
H40	0.8134	0.9720	0.1921	0.086*	
C22	0.9479 (2)	0.8018 (3)	0.02791 (18)	0.0749 (7)	
H41	0.9919	0.8095	−0.0218	0.090*	

### Atomic displacement parameters (Å^2^)

**Table:**

	*U*^11^	*U*^22^	*U*^33^	*U*^12^	*U*^13^	*U*^23^
O3	0.0527 (8)	0.0584 (8)	0.0618 (8)	0.0254 (6)	0.0289 (6)	0.0291 (6)
C18	0.0498 (12)	0.1065 (19)	0.0677 (13)	0.0350 (12)	−0.0025 (10)	0.0316 (13)
N1	0.0486 (8)	0.0388 (7)	0.0373 (7)	0.0205 (6)	0.0142 (6)	0.0153 (6)
N2	0.0497 (8)	0.0463 (8)	0.0329 (7)	0.0160 (7)	0.0073 (6)	0.0124 (6)
N3	0.0395 (7)	0.0338 (7)	0.0356 (6)	0.0132 (6)	0.0134 (6)	0.0162 (5)
N4	0.0363 (7)	0.0493 (8)	0.0347 (7)	0.0142 (6)	0.0091 (6)	0.0175 (6)
O2	0.0397 (6)	0.0585 (7)	0.0406 (6)	0.0190 (6)	0.0023 (5)	0.0152 (6)
C8	0.0366 (8)	0.0386 (8)	0.0334 (7)	0.0155 (7)	0.0116 (6)	0.0159 (6)
C16	0.0383 (8)	0.0319 (7)	0.0309 (7)	0.0123 (6)	0.0096 (6)	0.0134 (6)
C29	0.0411 (9)	0.0349 (8)	0.0290 (7)	0.0101 (7)	0.0036 (6)	0.0085 (6)
O1	0.0659 (9)	0.0504 (8)	0.0650 (8)	0.0323 (7)	0.0122 (7)	0.0217 (6)
C6	0.0389 (8)	0.0398 (8)	0.0361 (8)	0.0115 (7)	0.0098 (7)	0.0189 (7)
C7	0.0386 (8)	0.0341 (8)	0.0295 (7)	0.0100 (7)	0.0078 (6)	0.0137 (6)
C9	0.0376 (8)	0.0428 (9)	0.0319 (7)	0.0159 (7)	0.0100 (6)	0.0158 (7)
C5	0.0388 (9)	0.0436 (9)	0.0366 (8)	0.0144 (7)	0.0110 (7)	0.0199 (7)
C26	0.0438 (9)	0.0344 (8)	0.0313 (7)	0.0101 (7)	0.0065 (7)	0.0122 (6)
C10	0.0443 (9)	0.0426 (9)	0.0386 (8)	0.0153 (8)	0.0113 (7)	0.0103 (7)
C17	0.0418 (9)	0.0435 (9)	0.0375 (8)	0.0170 (8)	0.0110 (7)	0.0187 (7)
C15	0.0456 (9)	0.0380 (9)	0.0424 (9)	0.0185 (7)	0.0159 (7)	0.0122 (7)
C19	0.0399 (9)	0.0427 (9)	0.0336 (8)	0.0104 (7)	0.0107 (7)	0.0154 (7)
C20	0.0378 (9)	0.0448 (9)	0.0430 (9)	0.0050 (7)	0.0060 (7)	0.0216 (8)
C30	0.0524 (10)	0.0393 (9)	0.0354 (8)	0.0184 (8)	0.0067 (7)	0.0098 (7)
C14	0.0693 (13)	0.0434 (10)	0.0568 (11)	0.0258 (9)	0.0209 (10)	0.0185 (9)
C1	0.0618 (12)	0.0389 (9)	0.0424 (9)	0.0063 (8)	0.0066 (8)	0.0173 (8)
C32	0.0536 (11)	0.0528 (10)	0.0340 (8)	0.0106 (9)	0.0091 (8)	0.0144 (8)
C27	0.0408 (9)	0.0546 (10)	0.0367 (8)	0.0071 (8)	0.0062 (7)	0.0144 (8)
C34	0.0536 (11)	0.0544 (10)	0.0393 (9)	0.0273 (9)	0.0119 (8)	0.0198 (8)
C31	0.0548 (11)	0.0534 (10)	0.0367 (9)	0.0214 (9)	0.0133 (8)	0.0084 (8)
C33	0.0632 (12)	0.0584 (11)	0.0416 (9)	0.0249 (10)	0.0084 (8)	0.0258 (8)
C11	0.0606 (12)	0.0542 (11)	0.0445 (10)	0.0162 (10)	0.0073 (9)	0.0066 (9)
C4	0.0564 (11)	0.0565 (11)	0.0405 (9)	0.0173 (9)	0.0104 (8)	0.0282 (8)
C25	0.0723 (13)	0.0439 (10)	0.0518 (10)	0.0129 (9)	0.0156 (10)	0.0215 (9)
C3	0.0661 (12)	0.0561 (11)	0.0572 (11)	0.0153 (10)	0.0129 (10)	0.0383 (10)
C21	0.0503 (11)	0.0746 (13)	0.0676 (12)	0.0185 (10)	0.0235 (10)	0.0445 (11)
C28	0.0435 (10)	0.0802 (14)	0.0545 (11)	0.0221 (10)	0.0193 (9)	0.0257 (10)
C23	0.0649 (14)	0.0693 (15)	0.1019 (18)	0.0036 (12)	0.0038 (13)	0.0620 (14)
C12	0.0666 (14)	0.0476 (11)	0.0629 (13)	0.0151 (10)	0.0100 (11)	0.0009 (10)
C13	0.0742 (14)	0.0390 (10)	0.0765 (14)	0.0203 (10)	0.0232 (12)	0.0127 (10)
C2	0.0725 (14)	0.0425 (10)	0.0594 (12)	0.0079 (9)	0.0078 (10)	0.0280 (9)
C35	0.0943 (18)	0.0819 (15)	0.0520 (12)	0.0213 (13)	0.0321 (12)	0.0311 (11)
C24	0.0872 (16)	0.0458 (11)	0.0793 (15)	0.0158 (11)	0.0111 (13)	0.0309 (11)
C22	0.0581 (13)	0.0978 (18)	0.0932 (17)	0.0169 (13)	0.0233 (12)	0.0727 (16)

### Geometric parameters (Å, º)

**Table:**

O3—C19	1.2184 (19)	C30—H21	0.9300
C18—O2	1.438 (2)	C14—C13	1.363 (3)
C18—H1A	0.9600	C14—H22	0.9300
C18—H1B	0.9600	C1—C2	1.385 (2)
C18—H1C	0.9600	C1—H23	0.9300
N1—C8	1.3013 (19)	C32—C33	1.376 (3)
N1—C15	1.380 (2)	C32—C31	1.379 (3)
N2—C9	1.3085 (19)	C32—C35	1.509 (2)
N2—C10	1.370 (2)	C27—H25A	0.9700
N3—C19	1.3490 (19)	C27—H25B	0.9700
N3—C16	1.4458 (18)	C34—C33	1.380 (2)
N3—H3	0.8600	C34—H26	0.9300
N4—C28	1.451 (2)	C31—H27	0.9300
N4—C27	1.457 (2)	C33—H28	0.9300
N4—C7	1.460 (2)	C11—C12	1.356 (3)
O2—C17	1.3356 (19)	C11—H29	0.9300
C8—C9	1.424 (2)	C4—C3	1.370 (3)
C8—C7	1.520 (2)	C4—H30	0.9300
C16—C17	1.524 (2)	C25—C24	1.381 (3)
C16—C26	1.563 (2)	C25—H31	0.9300
C16—C7	1.576 (2)	C3—C2	1.378 (3)
C29—C30	1.379 (2)	C3—H32	0.9300
C29—C34	1.382 (2)	C21—C22	1.379 (3)
C29—C26	1.510 (2)	C21—H33	0.9300
O1—C17	1.1929 (19)	C28—H34A	0.9600
C6—C1	1.377 (2)	C28—H34B	0.9600
C6—C5	1.395 (2)	C28—H34C	0.9600
C6—C7	1.543 (2)	C23—C22	1.370 (3)
C9—C5	1.450 (2)	C23—C24	1.370 (3)
C5—C4	1.386 (2)	C23—H35	0.9300
C26—C27	1.534 (2)	C12—C13	1.390 (3)
C26—H15	0.9800	C12—H36	0.9300
C10—C11	1.405 (2)	C13—H37	0.9300
C10—C15	1.414 (2)	C2—H38	0.9300
C15—C14	1.401 (2)	C35—H39A	0.9600
C19—C20	1.497 (2)	C35—H39B	0.9600
C20—C25	1.379 (3)	C35—H39C	0.9600
C20—C21	1.386 (2)	C24—H40	0.9300
C30—C31	1.385 (2)	C22—H41	0.9300
			
O2—C18—H1A	109.5	C15—C14—H22	119.9
O2—C18—H1B	109.5	C6—C1—C2	119.23 (16)
H1A—C18—H1B	109.5	C6—C1—H23	120.4
O2—C18—H1C	109.5	C2—C1—H23	120.4
H1A—C18—H1C	109.5	C33—C32—C31	117.81 (16)
H1B—C18—H1C	109.5	C33—C32—C35	120.83 (18)
C8—N1—C15	114.78 (13)	C31—C32—C35	121.35 (18)
C9—N2—C10	114.47 (13)	N4—C27—C26	106.32 (13)
C19—N3—C16	123.49 (13)	N4—C27—H25A	110.5
C19—N3—H3	118.3	C26—C27—H25A	110.5
C16—N3—H3	118.3	N4—C27—H25B	110.5
C28—N4—C27	112.91 (14)	C26—C27—H25B	110.5
C28—N4—C7	116.08 (13)	H25A—C27—H25B	108.7
C27—N4—C7	108.12 (12)	C33—C34—C29	121.44 (16)
C17—O2—C18	115.42 (15)	C33—C34—H26	119.3
N1—C8—C9	122.96 (14)	C29—C34—H26	119.3
N1—C8—C7	126.40 (13)	C32—C31—C30	121.19 (16)
C9—C8—C7	110.64 (13)	C32—C31—H27	119.4
N3—C16—C17	111.13 (12)	C30—C31—H27	119.4
N3—C16—C26	112.04 (11)	C32—C33—C34	121.02 (16)
C17—C16—C26	112.59 (12)	C32—C33—H28	119.5
N3—C16—C7	107.65 (11)	C34—C33—H28	119.5
C17—C16—C7	110.42 (11)	C12—C11—C10	120.40 (19)
C26—C16—C7	102.58 (11)	C12—C11—H29	119.8
C30—C29—C34	117.49 (14)	C10—C11—H29	119.8
C30—C29—C26	120.39 (14)	C3—C4—C5	118.55 (16)
C34—C29—C26	122.01 (14)	C3—C4—H30	120.7
C1—C6—C5	119.04 (14)	C5—C4—H30	120.7
C1—C6—C7	130.03 (14)	C20—C25—C24	120.62 (19)
C5—C6—C7	110.92 (13)	C20—C25—H31	119.7
N4—C7—C8	112.45 (12)	C24—C25—H31	119.7
N4—C7—C6	117.15 (12)	C4—C3—C2	120.44 (16)
C8—C7—C6	100.68 (11)	C4—C3—H32	119.8
N4—C7—C16	99.76 (11)	C2—C3—H32	119.8
C8—C7—C16	114.22 (12)	C22—C21—C20	119.6 (2)
C6—C7—C16	113.29 (12)	C22—C21—H33	120.2
N2—C9—C8	123.90 (14)	C20—C21—H33	120.2
N2—C9—C5	127.69 (14)	N4—C28—H34A	109.5
C8—C9—C5	108.40 (13)	N4—C28—H34B	109.5
C4—C5—C6	121.54 (15)	H34A—C28—H34B	109.5
C4—C5—C9	129.34 (15)	N4—C28—H34C	109.5
C6—C5—C9	109.11 (13)	H34A—C28—H34C	109.5
C29—C26—C27	115.40 (13)	H34B—C28—H34C	109.5
C29—C26—C16	113.57 (12)	C22—C23—C24	120.20 (19)
C27—C26—C16	103.91 (12)	C22—C23—H35	119.9
C29—C26—H15	107.9	C24—C23—H35	119.9
C27—C26—H15	107.9	C11—C12—C13	120.56 (19)
C16—C26—H15	107.9	C11—C12—H36	119.7
N2—C10—C11	119.22 (16)	C13—C12—H36	119.7
N2—C10—C15	121.76 (14)	C14—C13—C12	120.73 (19)
C11—C10—C15	119.02 (16)	C14—C13—H37	119.6
O1—C17—O2	124.37 (15)	C12—C13—H37	119.6
O1—C17—C16	125.32 (15)	C3—C2—C1	121.18 (17)
O2—C17—C16	110.03 (13)	C3—C2—H38	119.4
N1—C15—C14	119.11 (15)	C1—C2—H38	119.4
N1—C15—C10	121.81 (14)	C32—C35—H39A	109.5
C14—C15—C10	119.05 (15)	C32—C35—H39B	109.5
O3—C19—N3	122.17 (14)	H39A—C35—H39B	109.5
O3—C19—C20	122.11 (14)	C32—C35—H39C	109.5
N3—C19—C20	115.72 (14)	H39A—C35—H39C	109.5
C25—C20—C21	119.33 (16)	H39B—C35—H39C	109.5
C25—C20—C19	122.78 (15)	C23—C24—C25	119.6 (2)
C21—C20—C19	117.89 (16)	C23—C24—H40	120.2
C29—C30—C31	121.02 (16)	C25—C24—H40	120.2
C29—C30—H21	119.5	C23—C22—C21	120.6 (2)
C31—C30—H21	119.5	C23—C22—H41	119.7
C13—C14—C15	120.19 (18)	C21—C22—H41	119.7
C13—C14—H22	119.9		
			
C15—N1—C8—C9	5.3 (2)	C9—N2—C10—C15	3.4 (2)
C15—N1—C8—C7	−174.39 (14)	C18—O2—C17—O1	2.3 (2)
C19—N3—C16—C17	39.35 (18)	C18—O2—C17—C16	176.53 (15)
C19—N3—C16—C26	−87.57 (17)	N3—C16—C17—O1	−131.07 (16)
C19—N3—C16—C7	160.39 (13)	C26—C16—C17—O1	−4.4 (2)
C28—N4—C7—C8	−67.27 (17)	C7—C16—C17—O1	109.54 (17)
C27—N4—C7—C8	164.69 (12)	N3—C16—C17—O2	54.81 (16)
C28—N4—C7—C6	48.66 (19)	C26—C16—C17—O2	−178.56 (12)
C27—N4—C7—C6	−79.37 (15)	C7—C16—C17—O2	−64.57 (16)
C28—N4—C7—C16	171.29 (14)	C8—N1—C15—C14	177.13 (15)
C27—N4—C7—C16	43.26 (14)	C8—N1—C15—C10	−1.0 (2)
N1—C8—C7—N4	−50.7 (2)	N2—C10—C15—N1	−3.6 (3)
C9—C8—C7—N4	129.51 (13)	C11—C10—C15—N1	175.92 (15)
N1—C8—C7—C6	−176.22 (15)	N2—C10—C15—C14	178.32 (16)
C9—C8—C7—C6	4.03 (16)	C11—C10—C15—C14	−2.2 (2)
N1—C8—C7—C16	62.0 (2)	C16—N3—C19—O3	−4.5 (2)
C9—C8—C7—C16	−117.71 (14)	C16—N3—C19—C20	175.11 (13)
C1—C6—C7—N4	55.1 (2)	O3—C19—C20—C25	−153.06 (18)
C5—C6—C7—N4	−123.65 (14)	N3—C19—C20—C25	27.3 (2)
C1—C6—C7—C8	177.36 (17)	O3—C19—C20—C21	27.2 (2)
C5—C6—C7—C8	−1.41 (16)	N3—C19—C20—C21	−152.45 (16)
C1—C6—C7—C16	−60.2 (2)	C34—C29—C30—C31	1.7 (2)
C5—C6—C7—C16	120.99 (14)	C26—C29—C30—C31	−174.49 (15)
N3—C16—C7—N4	78.00 (13)	N1—C15—C14—C13	−175.62 (17)
C17—C16—C7—N4	−160.52 (12)	C10—C15—C14—C13	2.6 (3)
C26—C16—C7—N4	−40.32 (13)	C5—C6—C1—C2	1.5 (3)
N3—C16—C7—C8	−42.15 (16)	C7—C6—C1—C2	−177.21 (17)
C17—C16—C7—C8	79.32 (15)	C28—N4—C27—C26	−158.68 (14)
C26—C16—C7—C8	−160.48 (12)	C7—N4—C27—C26	−28.85 (16)
N3—C16—C7—C6	−156.67 (12)	C29—C26—C27—N4	−123.90 (14)
C17—C16—C7—C6	−35.19 (17)	C16—C26—C27—N4	1.11 (16)
C26—C16—C7—C6	85.01 (14)	C30—C29—C34—C33	−2.2 (3)
C10—N2—C9—C8	0.9 (2)	C26—C29—C34—C33	173.90 (16)
C10—N2—C9—C5	−179.94 (15)	C33—C32—C31—C30	−1.6 (3)
N1—C8—C9—N2	−5.7 (2)	C35—C32—C31—C30	176.85 (18)
C7—C8—C9—N2	174.02 (14)	C29—C30—C31—C32	0.2 (3)
N1—C8—C9—C5	174.98 (14)	C31—C32—C33—C34	1.1 (3)
C7—C8—C9—C5	−5.26 (17)	C35—C32—C33—C34	−177.38 (18)
C1—C6—C5—C4	−1.6 (2)	C29—C34—C33—C32	0.9 (3)
C7—C6—C5—C4	177.35 (15)	N2—C10—C11—C12	179.90 (18)
C1—C6—C5—C9	179.45 (15)	C15—C10—C11—C12	0.4 (3)
C7—C6—C5—C9	−1.63 (18)	C6—C5—C4—C3	0.4 (3)
N2—C9—C5—C4	6.1 (3)	C9—C5—C4—C3	179.20 (17)
C8—C9—C5—C4	−174.62 (17)	C21—C20—C25—C24	−1.6 (3)
N2—C9—C5—C6	−174.99 (15)	C19—C20—C25—C24	178.65 (18)
C8—C9—C5—C6	4.25 (18)	C5—C4—C3—C2	0.8 (3)
C30—C29—C26—C27	−144.82 (16)	C25—C20—C21—C22	0.8 (3)
C34—C29—C26—C27	39.2 (2)	C19—C20—C21—C22	−179.47 (17)
C30—C29—C26—C16	95.34 (17)	C10—C11—C12—C13	1.1 (3)
C34—C29—C26—C16	−80.66 (19)	C15—C14—C13—C12	−1.1 (3)
N3—C16—C26—C29	35.05 (17)	C11—C12—C13—C14	−0.8 (3)
C17—C16—C26—C29	−91.08 (15)	C4—C3—C2—C1	−0.8 (3)
C7—C16—C26—C29	150.23 (12)	C6—C1—C2—C3	−0.3 (3)
N3—C16—C26—C27	−91.12 (14)	C22—C23—C24—C25	0.5 (3)
C17—C16—C26—C27	142.74 (13)	C20—C25—C24—C23	1.0 (3)
C7—C16—C26—C27	24.05 (14)	C24—C23—C22—C21	−1.3 (4)
C9—N2—C10—C11	−176.11 (15)	C20—C21—C22—C23	0.7 (3)

### Hydrogen-bond geometry (Å, º)

**Table:**

*D*—H···*A*	*D*—H	H···*A*	*D*···*A*	*D*—H···*A*
N3—H3···N1	0.86	2.27	2.8107 (18)	121
C21—H33···O3^i^	0.93	2.54	3.347 (2)	146

Symmetry code: (i) −*x*+2, −*y*+1, −*z*.
